# Non-inferiority test for a continuous variable with a flexible margin in an active controlled trial: an application to the “Stratall ANRS 12110 / ESTHER” trial

**DOI:** 10.1186/s13063-022-06118-x

**Published:** 2022-03-05

**Authors:** Arsene Brunelle Sandie, Nicolas Molinari, Anthony Wanjoya, Charles Kouanfack, Christian Laurent, Jules Brice Tchatchueng-Mbougua

**Affiliations:** 1 African Population and Health Research Center - West Africa Regional Office, Dakar, Senegal; 2 IMAG,CNRS, Université Montpellier, CHU Montpellier, Montpellier, France; 3 Department of Statistics and Actuarial Sciences, Jomo Kenyatta University of Agriculture and Technology (JKUAT), Nairobi, Kenya; 4 Day Hospital, Yaounde Central Hospital, Yaounde, Cameroon; 5 IRD, INSERM, Université Montpellier, TransVIHMI, Montpellier, France; 6 Université de Yaoundé I - CETIC, UPMC Université Paris 06, IRD, Unité de Modélisation Mathématique et Informatique des Systèmes Complexes (UMMISCO), Bondy, France

**Keywords:** Asymptotic test, Active controlled trial, Confidence interval, Flexible margin, Non-inferiority

## Abstract

**Background:**

Non-inferiority trials are becoming increasingly popular in public health and clinical research. The choice of the non-inferiority margin is the cornerstone of such trials. Most of the time, the non-inferiority margin is fixed and constant, determined from historical trials as a fraction of the effect of the reference intervention. But in some circumstances, there may some uncertainty around the reference treatment that one would like to account for when performing the hypothesis testing. In this case, the non-inferiority margin is not fixed in advance and depends on the reference intervention estimate. Hence, the uncertainty surrounding the non-inferiority margin should be accounted for in statistical tests. In this work, we explore how to perform the non-inferiority test for a continuous variable with a flexible margin.

**Methods:**

We have proposed in this study, two procedures for the non-inferiority test with a flexible margin for continuous endpoints. The proposed test procedures are based on a test statistic and confidence interval approaches respectively. Simulations have been used to assess the performances and properties of the proposed test procedures. An application was done on a real-world clinical data, to assess the efficacy of clinical monitoring alone versus laboratory and clinical monitoring in HIV-infected adult patients.

**Results:**

Basically, for both proposed methods, the type I error estimate was not dependent on the values of the reference treatment. In the test statistic approach, the type 1 error rate estimate was approximatively equal to the nominal value. It has been found that the confidence interval level determined approximatively the level of significance. For a given nominal type I error *α*, the appropriate one- and two-sided confidence intervals should be with levels 1−*α* and 1−2*α*, respectively.

**Conclusions:**

Based on the type I error rate and power estimates, the proposed non-inferiority hypothesis test procedures had good performances and were applicable in practice.

**Trial registration:**

ClinicalTrials.gov NCT00301561. Registered on March 13, 2006, url: https://clinicaltrials.gov/ct2/show/NCT00301561.

## Background

After developing a new health intervention (treatment or diagnostic test), the next step is to assess its effectiveness, relative to the existing reference intervention. There are several strategies to do this, such as the superiority trials which involve testing whether the new treatment is superior to another (placebo, reference, or active control treatment). However, when the active control intervention achieves maximum efficacy or the use of a placebo is unethical, it becomes difficult to statistically show the superiority of the new health intervention. Studies aimed at showing that a new intervention is not worse than the active control intervention by more than a pre-specified amount of efficacy have become increasingly common in the recent decade [1]. The expression *is not worse than the active control intervention by more than a pre-specified amount*, means it is acceptable to lose a “little bit” of the main effect of the active control intervention compared to a new intervention’s benefits (fewer side effects, costs, tolerable, and safer). This acceptable loss of efficacy is illustrated numerically as the non-inferiority margin. A trial showing that the new intervention is *non-inferior* to the active control intervention is called *a non-inferiority trial* [1].

The Food and Drug Administration (FDA)[2] provided general principles for an appropriate choice of the non-inferiority margin. The non-inferiority margin is at the upper limit of the confidence interval, so the trial is designed to show evidence of no more than this “loss of maximum efficacy.” Generally, this margin is fixed, determined from historical trials as a fraction of the treatment effect. However, in some cases, the mean estimate of reference treatment could be subjected to variations to the levels that adopting a fixed margin would not be relevant. Indeed, the fixed margin cannot take into account the variability which surrounds the reference treatment estimate, in this case, the margin should be a function of the reference treatment. For binary endpoints, tests that account for non-fixed margins have been studied [3–5]. One finds that most works on the non-inferiority test for continuous endpoints with fixed and linear margin have been focused on the confidence intervals approach [6–8], mainly consisting of comparing the bounds of the treatments difference to the fixed margin. However, few studies have been performed for a non-fixed or variable margin for continuous endpoints. This work is aimed at deriving non-inferiority tests for continuous endpoints with flexible margin in active randomized controlled trials. An application of the proposed methods is done on the Stratall ANRS 12110/ESTHER trial.

## Methods

### Notations

The following are the definition of the basic notations used.

- *X*_*R*_ and *X*_*N*_ are the the random variables for continuous primary endpoint in the active control group (reference) and new intervention group (new group), respectively.
- *n*_*R*_ and *n*_*N*_ are the the sample sizes for the active control group and new group, respectively.
- *μ*_*R*_ and *μ*_*N*_ are the the means of continuous primary endpoint for the active group and new group, respectively.
- ${\sigma }^{2}_{R}$ and ${\sigma }^{2}_{N}$ are the the variances of continuous primary endpoint for the active group and new group respectively.
- *Δ*_*L*_(*μ*_*R*_) is the non-inferiority margin, and *Δ*=*μ*_*N*_−*μ*_*R*_ is the difference of true means.
- *H*_0_ and *H*_1_ are the null and alternative hypotheses, respectively.

### Approach using a test statistic

Without loss of generality, assuming that an increase in the endpoint corresponds to more efficacy. The non-inferiority hypotheses can be formulated as follows:

1 $$ \left \{ \begin{array}{ll} H_{0}{:} \mu_{N} \leq \mu_{R}-\Delta_{L} & \mathrm{There is no non-inferiority}\\ H_{1}{:} \mu_{N} > \mu_{R}-\Delta_{L} & \mathrm{There is non-inferiority} \end{array} \right.  $$

The formulation of the hypotheses test in Eq. (1) shows that the non-inferiority means that the new intervention is not worse than the active control intervention with a *Δ*_*L*_ margin. When *Δ*_*L*_ is fixed, testing the hypotheses (1) can be viewed as a classical composite hypotheses test for mean difference [9]; therefore, based on the central limit theorem applied to the boundary of the null hypothesis, the asymptotic test *Z*_fixed_ can be obtained by:

2 $$  Z_{\text{fixed}}=\frac{\bar{X}_{N}-\bar{X}_{R}+\Delta_{L}}{\sqrt{\frac{{\sigma}^{2}_{N}}{n_{N}}+\frac{{\sigma}^{2}_{R}}{n_{R}}}}\sim N(0,1).  $$

In effect, when *Δ*_*L*_ is fixed, we have:

3 $$\begin{array}{*{20}l} \text{Var}(\bar{X}_{N}-\bar{X}_{R}+\Delta_{L}) &=\text{Var}(\bar{X}_{N})+\text{Var}(\bar{X}_{R}) \notag \\ &=\frac{{\sigma}^{2}_{N}}{n_{N}}+\frac{{\sigma}^{2}_{R}}{n_{R}}. \end{array} $$

The null hypothesis is rejected if *Z*_fixed_>*Z*_1−*α*_, where *Z*_1−*α*_ is the (1−*α*) percentile of the standard normal distribution. From the Karlin-Rubin theorem, this test is the uniformly most powerful test of level *α* [10].

If *Δ*_*L*_ is not fixed, i.e, if *Δ*_*L*_ is a function of *μ*_*R*_, then $\text {Var}\{\bar {X}_{N}-\bar {X}_{R}+\Delta _{L}(\bar {X}_{R})\}\neq \text {Var}(\bar {X}_{N})+\text {Var}(\bar {X}_{R})$, and therefore, $\text {Var}(\bar {X}_{N})+\text {Var}(\bar {X}_{R})$ is not a valid variance of $\bar {X}_{N}-\bar {X}_{R}+\Delta _{L}(\bar {X}_{R})$. Under the assumption that *Δ*_*L*_ is a continuously differentiable function, variance estimation was performed using delta method discussed below.

#### Variance estimation using delta method

If *Δ*_*L*_(.) is a continuously differentiable such that *ΔL*′(*μ*_*R*_)≠0 (*ΔL*′ is the first derivative of *Δ*_*L*_), then using the Taylor series of order 1 in a neighborhood of *μ*_*R*_,

4 $$ \Delta_{L}(\bar{X}_{R})=\Delta_{L}(\mu_{R})+\Delta'_{L}(\mu_{R})(\bar{X}_{R}-\mu_{R})+o_{p}(1).  $$

Hence,

$$\begin{array}{*{20}l} &{}\{\bar{X}_{N}-\bar{X}_{R}+\Delta_{L}(\bar{X}_{R})\}-\{\mu_{N}-\mu_{R}+\Delta_{L}(\mu_{R})\}\\ &{}=(\bar{X}_{N}-\mu_{N})-(\bar{X}_{R}-\mu_{R})+\{\Delta_{L}(\bar{X}_{R})-\Delta_{L}(\mu_{R})\}\\ &{}=(\bar{X}_{N}-\mu_{N})-(\bar{X}_{R}-\mu_{R})+\Delta'_{L}(\mu_{R})(\bar{X}_{R}-\mu_{R})+o_{p}(1)\\ &{}=(\bar{X}_{N}-\mu_{N})+\{\Delta'_{L}(\mu_{R})-1\}(\bar{X}_{R}-\mu_{R})+o_{p}(1)\\ \end{array} $$

Thus, the variance estimate is:

5 $$ {}\text{Var}\{\bar{X}_{N}-\bar{X}_{R}+\Delta_{L}(\bar{X}_{R})\} = \frac{\sigma^{2}_{N}}{n_{N}}+\frac{\{\Delta'_{L}(\mu_{R})-1\}^{2}\sigma^{2}_{R}}{n_{R}}  $$

The test statistic can then be expressed as:

6 $$ {}Z_{\text{flexible}}=\frac{\{\bar{X}_{N}-\bar{X}_{R}+\Delta_{L}(\bar{X}_{R})\}-\{\mu_{N}-\mu_{R}+\Delta_{L}(\mu_{R})\}}{\sqrt{\frac{\sigma^{2}_{N}}{n_{N}}+\frac{\{\Delta'_{L}(\mu_{R})-1\}^{2}\sigma^{2}_{R}}{n_{R}}}}.  $$

#### Asymptotic properties of the test statistic *Z*_flexible_

From the central limit theorem, when *n*_*N*_ and *n*_*R*_ approach infinity, the random variable *Z*_flexible_∼*N*(0,1) on the boundary of null hypothesis, that is, asymptotically,

7 $$ Z_{\text{flexible}}=\frac{\bar{X}_{N}-\bar{X}_{R}+\Delta_{L}(\bar{X}_{R})}{\sqrt{\frac{\sigma^{2}_{N}}{n_{N}}+\frac{\{\Delta'_{L}(\mu_{R})-1\}^{2}\sigma^{2}_{R}}{n_{R}}}} \sim N(0, 1).  $$

*μ*_*R*_ is unknown and $\sigma ^{2}_{R}$ and $\sigma ^{2}_{N}$ may be unknowns, which need to be estimated. We used the maximum likelihood estimation method on the boundary of the null hypothesis (*μ*_*N*_=*μ*_*R*_−*Δ*_*L*_(*μ*_*R*_)). The unknown parameters are estimated considering the cases where the variances $\sigma ^{2}_{R}$ and $\sigma ^{2}_{N}$ are known, unknown, equal, or unequal.

The maximum likelihood (ML) estimators $\hat {\mu _{R}}, \hat {\sigma _{R}}^{2}$ and $\hat {\sigma _{N}}^{2}$ for $\mu _{R}, \sigma ^{2}_{R}$ and $\sigma ^{2}_{N}$, respectively, are consistent. Moreover, since *ΔL*′ is assumed continuous, $\Delta '_{L}(\hat {\mu _{R}})$ is a consistent estimator for *ΔL*′(*μ*_*R*_). The estimator $\hat {Z}_{\text {flexible}}$ of the test statistic *Z*_flexible_ can be obtained by replacing the unknown parameters in (6) by their ML estimators. Therefore, the test *H*0′ versus *H*_1_ (where *H*0′ is the boundary of *H*_0_ i.e *μ*_*N*_=*μ*_*R*_−*Δ*_*L*_(*μ*_*R*_)) is rejected if $\hat {Z}_{\text {flexible}}>z_{1-\alpha }$, where *α* is the nominal type I error and *z*_1−*α*_ denotes the 1−*α* percentile of the standard normal distribution. The significance level of this test tends to *α* when *n*_*N*_ and *n*_*R*_ approach infinity.

Assuming that, under alternative hypotheses *H*_1_,*μ*_*N*_−*μ*_*R*_+*Δ*_*L*_(*μ*_*R*_)=*v*, we have *v*>0. Hence, if *η* is the power of the test, it follows that:

$$\begin{array}{*{20}l} \eta &= \mathbf{P}\left(\frac{\bar{X}_{N}-\bar{X}_{R}+\Delta_{L}(\bar{X}_{R})}{\sqrt{\frac{\sigma^{2}_{N}} {n_{N}}+\frac{(\Delta'_{L}(\mu_{R})-1)^{2}\sigma^{2}_{R}}{n_{R}}}} > z_{1-\alpha} /H_{1}\right)\\ & = \mathbf{P}\left(\frac{\bar{X}_{N}-\bar{X}_{R}+\Delta_{L}(\bar{X}_{R})-v}{\sqrt{\frac{\sigma^{2}_{N}} {n_{N}}+\frac{(\Delta'_{L}(\mu_{R})-1)^{2}\sigma^{2}_{R}}{n_{R}}}} \right. \\  &>\left. z_{1-\alpha}-\frac{v}{\sqrt{\frac{\sigma^{2}_{N}} {n_{N}}+\frac{(\Delta'_{L}(\mu_{R})-1)^{2}\sigma^{2}_{R}}{n_{R}}}}\right), \end{array} $$

where, under alternative hypothesis, $\frac {\bar {X}_{N}-\bar {X}_{R}+\Delta _{L}(\bar {X}_{R})-v}{\sqrt {\frac {\sigma ^{2}_{N}} {n_{N}}+\frac {(\Delta '_{L}(\mu _{R})-1)^{2}\sigma ^{2}_{R}}{n_{R}}}} \sim N(0,1)$. Assuming the equal variance in both groups ($\sigma ^{2} = \sigma ^{2}_{R} =\sigma ^{2}_{N}$) and denoting by *δ*=*v*/*σ*, the power, given as a function of *δ*,*n*_*N*_,*n*_*R*_, and *α* is:

8 $$  \eta(\delta, n_{N}, n_{R})=\Phi\left(\frac{\delta}{\sqrt{\frac{1} {n_{N}}+\frac{(\Delta'_{L}(\mu_{R})-1)^{2}}{n_{R}}}}-z_{1-\alpha}\right),  $$

where *Φ* is the cumulative distribution function of the standard normal distribution. For a fixed nominal type I error *α*, and for any fixed *μ*_*R*_ and *μ*_*N*_ such that *v*=*μ*_*N*_−*μ*_*R*_+*Δ*_*L*_(*μ*_*R*_)>0, when *n*_*R*_→*∞* and *n*_*N*_→*∞*, it follows that *η*→1. Therefore, the test *Z*_flexible_ is asymptotically convergent. From Eq. 8, it is possible to find the sample size that achieves the nominal fixed power. Denoting the nominal type II error by *β* and assuming that *n*_*N*_=*rn*_*R*_ with *r*>0, the sample size which will allow nominal power (1−*β*) is such that:

9 $$ n_{R} \geq \frac{(z_{1-\alpha}+z_{1-\beta})^{2}\left[1+r\{\Delta'_{L}(\mu_{R})-1\}^{2}\right]}{r\delta^{2}}.  $$

This formula is equivalent to the one found in [9] when the margin is fixed. Practically, *δ* is equivalent to the standardized difference in the comparison of the means, and in this work, it would be named *standardized non-inferiority difference*. In the power and sample sizes calculations, one will fix *δ* (for example, *δ*=0.05 or *δ*=0.5 if one wants to detect small or large inferiority differences respectively), and *μ*_*R*_ could be pre-specified from historical studies with similar treatment.

The proposed test statistic $\hat {Z}_{\text {flexible}}$ is asymptotic, hence works well for large sample sizes, hence not adapted for datasets with small sample sizes, which are not uncommon in pratical situations. In such cases, the non-parametric test based on the percentile bootstrap confidence interval which does not require any assumptions on the sample size or sample distribution can be used[11].

### Approach based on confidence intervals

For any test based on confidence intervals, the main interest is on the level of confidence intervals which is required to achieve a desired nominal type I error. Moreover, as discussed in [9] and [12], the type I error is a controversial issue in clinical trial tests. In the framework of non-inferiority tests, when the non-inferiority margin is fixed, [13] recommended using 1−*α* and $1-\frac {\alpha }{2}$ for two-sided and one-sided confidence interval levels respectively, while [7] recommended to use 1−2*α* for two-sided and 1−*α* for one-sided confidence intervals. In [7], it is argued that the recommendation of [13] would lead to a conservative test, as the estimate type I error rate would be half the nominal one. Moreover, it has been argued that there would be approximately a 10% loss of power. In this section, we propose a non-parametric procedure for the confidence interval (one-sided and two sided) construction when the non-inferiority margin is flexible.

An intuitive procedure based on confidence intervals for the hypotheses test in Eq. (1) would be by checking the overlapping of the confidence intervals of *μ*_*N*_−*μ*_*R*_ and −*Δ*_*L*_(*μ*_*R*_). The null hypothesis would be rejected if the two confidence intervals are non-overlapped and not rejected otherwise. In such case, as illustrated in [14], the intervals may be overlapped while the statistics would not be necessarily non-significantly different; thus, the power of the test would be lower. The proposed procedure involves comparing the lower bound of the confidence interval (one- or two-sided, respectively) with *γ%* level of *μ*_*N*_−*μ*_*R*_+*Δ*_*L*_(*μ*_*R*_) with 0. The null hypothesis *H*_0_ is rejected if the lower bound of the confidence interval for *μ*_*N*_−*μ*_*R*_+*Δ*_*L*_(*μ*_*R*_) is greater than 0.

Estimation of the type I error is performed using simulations and non-parametric estimation of confidence intervals on the boundary of the null hypothesis. The detailed steps are described below. 1. From a fixed *μ*_*R*_, calculate *μ*_*N*_=*μ*_*R*_−*Δ*_*L*_(*μ*_*R*_) (satisfying the null hypothesis *H*_0_). We assume that the standard deviations *σ*_*N*_ and *σ*_*R*_ are known. 2. Let *m* denote the number of desired simulations, for *i*∈{1⋯*m*}, simulate *m* pairs of samples *X*_*N*_ and *X*_*R*_ of size *n*_*N*_ and *n*_*R*_, respectively, from the normal distribution $\mathcal {N}(\mu _{N}, \sigma _{N})$ and $\mathcal {N}(\mu _{R}, \sigma _{R}),$ respectively. 3. Using bootstrap, compute the empirical percentile confidence intervals [*a*_*i*_,*∞*] for one-sided confidence interval (and [*a*_*i*_,*b*_*i*_] for two-sided confidence interval, respectively) of level *γ* for *μ*_*N*_−*μ*_*R*_+*Δ*_*L*_(*μ*_*R*_), for *i*∈{1⋯*m*}. 4. For *i*∈{1⋯*m*}*H*_0_ is rejected when *a*_*i*_>0, thus the level of significance is estimated by: $\alpha (\gamma)=\frac {1}{m}\sum ^{m}_{i=1}1_{a_{i}>0}$.

Like any other power estimation, the data are drawn under the alternative hypothesis that is, *μ*_*N*_>*μ*_*R*_−*Δ*_*L*_(*μ*_*R*_). Since there is a wide range of possibilities on the alternative hypothesis, in practice, one considers the equivalence point, that is, *μ*_*R*_=*μ*_*N*_. Therefore, similarly to studies of [5] and [15], the equivalence point (*μ*_*R*_=*μ*_*N*_) will be used for drawing data for the power estimation. 1. Given *μ*_*R*_, simulate *m* pairs of samples *X*_*N*_ and *X*_*R*_ of respective sizes *n*_*N*_ and *n*_*R*_ using the respective normal distributions $\mathcal {N}(\mu _{R}, \sigma _{N})$ and $\mathcal {N}(\mu _{R}, \sigma _{R})$. 2. Using bootstrap, compute the empirical percentile confidence intervals [*a*_*i*_,*b*_*i*_] of level *γ* for *μ*_*N*_−*μ*_*R*_+*Δ*_*L*_(*μ*_*R*_), for *i*∈{1⋯*m*}. 3. For *i*∈{1⋯*m*}*H*_0_ is rejected when *a*_*i*_>0. Thus, the power is estimated by, $\eta (\gamma)=\frac {1}{m}\sum ^{m}_{i=1}1_{a_{i}>0}$.

### Performances assessment

Simulations were done to evaluate the finite-sample performances of the asymptotic test and confidence interval based test. The performance indicators used were the type I error and statistical power. Monte-Carlo simulation techniques were used for the estimation of the considered indicators. In the simulations, we considered the margin $\Delta _{L}(\mu _{R})=\mu _{R}^{1/4}$; and unknown variances $\sigma _{R}^{2}$ and $\sigma _{N}^{2}$.

Both indicators were computed for the two proposed tests according to the reference treatment. For the type I error, data were drawn on the boundary of the null hypothesis: for a given *μ*_*R*_, *μ*_*N*_ is obtained such that *μ*_*N*_=*μ*_*R*_−*Δ*_*L*_(*μ*_*R*_). For the power, data were drawn under the alternative hypothesis: for a given *μ*_*R*_, *μ*_*N*_ is obtained such that *μ*_*N*_>*μ*_*R*_−*Δ*_*L*_(*μ*_*R*_). Usually, one takes *μ*_*N*_=*μ*_*R*_. In all cases, it is assumed that *μ*_*R*_ vary in [1,1000]. In the test based on statistic, the power was estimate using formula (8), and two cases were considered for *δ*=0.05 and *δ*=0.5.

In the approach based on the asymptotic test, the nominal type I error was fixed and set at *α*=5*%*. For the confidence interval based test, we considered 95% one- and two-sided confidence interval levels. The purpose was to estimate the type I error rate for the respective confidence interval. In all the simulations, we considered balanced sample sizes (that is when *n*=*n*_*N*_=*n*_*R*_), *n*=30,100, and 1000 for small, medium, and large sample sizes, respectively. The number of bootstrap samples with replacement was *B*=1000, and the number of simulation replications was *m*=10000. The **R** software programming language [16] was used to conduct the simulations and codes are accessible in a separate file on request.

### Application to the Stratall ANRS 12110 / ESTHER

This study was motivated by the randomized non-inferiority “Stratall ANRS 12110 / ESTHER” trial [17]. The main purpose was to assess an exclusively clinical monitoring strategy compared with a clinical monitoring strategy plus laboratory monitoring in terms of effectiveness and safety in HIV-infected patients in Cameroon. The idea was to achieve the scaling-up of HIV care in rural districts where most people live with HIV, but local health facilities generally have low-grade equipment. A total of 459 HIV-infected patients were included in the study and randomly allocated to two groups, one receiving exclusively clinical monitoring (intervention group, *N* = 238) and the other receiving laboratory and clinical monitoring (active control group (reference), *N* = 221). All patients included were initiated antiretroviral treatment and were followed up for 24 months. Clinical monitoring alone was compared to laboratory and clinical monitoring in a non-inferiority design. The continuous primary endpoint was the increase in CD4 cells count from treatment initiation to the twenty-fourth month. Based on previous studies, the non-inferiority margin (*Δ*_*L*_(*R*)) was prespecified as a linear function (25%) of the mean CD4 cells increase (*μ*_*R*_) after 24 months of antiretroviral treatment in laboratory and clinical monitoring group, $\Delta _{L}(R)= \frac {25}{100} \mu _{R}$. Unlike other non-inferiority studies [18, 19], the non-inferiority margin in this study was varied (depending on the mean increase in CD4 in the active control group (reference)). However, the classical two-sided confidence interval based test with 90% level were used to obtain a type I error (*α*) close to 5% [17]. Indeed, the statistical test procedures that explore the non-inferiority test for con- tinuous data with variable margins were not available at that time in the original paper [17]. Moreover, as discussed in [12], the relationship between the confidence intervals level and the type I error can be controversial.

More details about the background of the study and the clinical trial process can be found in [17]. Two analyses were done according to the type of data:

- Firstly, the increase of CD4 cells count at 24 months from the baseline was considered, which implies missing or lost patients before the end of follow-up period were excluded in the analysis. In that case, the total number of patient in the analysis reduced to *n*=334, with *n*_*R*_=169 and *n*_*N*_=165. “Observed data” will refer to the case where data are analyzed by excluding participants with missing observation at 24 months.
- Secondly, an analysis was done with all participants who attended at least one follow-up visit, and the last observation carried forward (LOCF) imputation method was applied for participants whose CD4 data were missing at 24 months (in this case, the number of patients to analyzed is the same as the baseline: *n*=459, *n*_*R*_=238, *n*_*N*_=221).

The classical parametric two-sided confidence interval based test with 90% level was used by [17] to perform the non-inferiority test. The final result was that the CLIN was inferior to the LAB.

## Results

### Simulations results

#### Test statistic based test

**Fig. 1:**
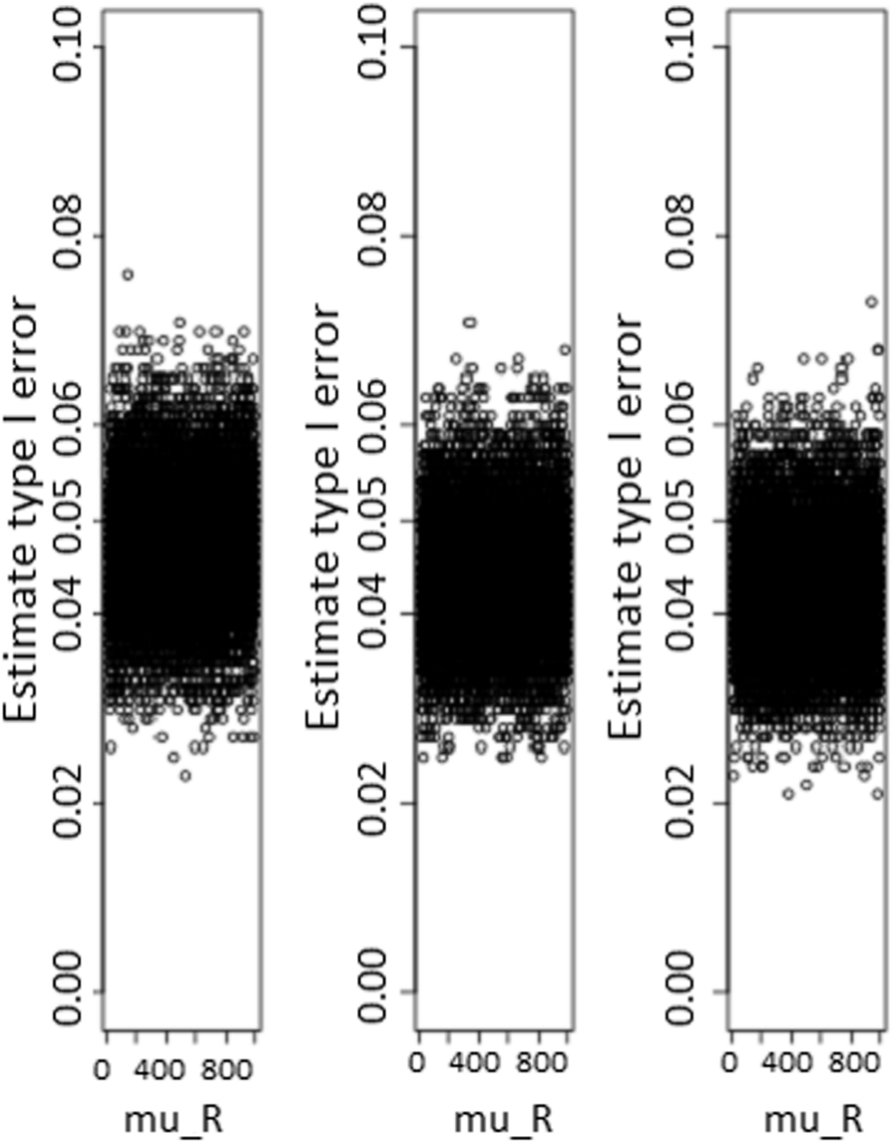
Type I error rate estimates according to sample sizes for test statistic based test. Type I error rate estimates as function of reference treatment, for the test statistic based test from the left to the rigth, sample sizes are *n*_*N*_=*n*_*R*_=20, 100, and 1000 respectively

**Fig. 2:**
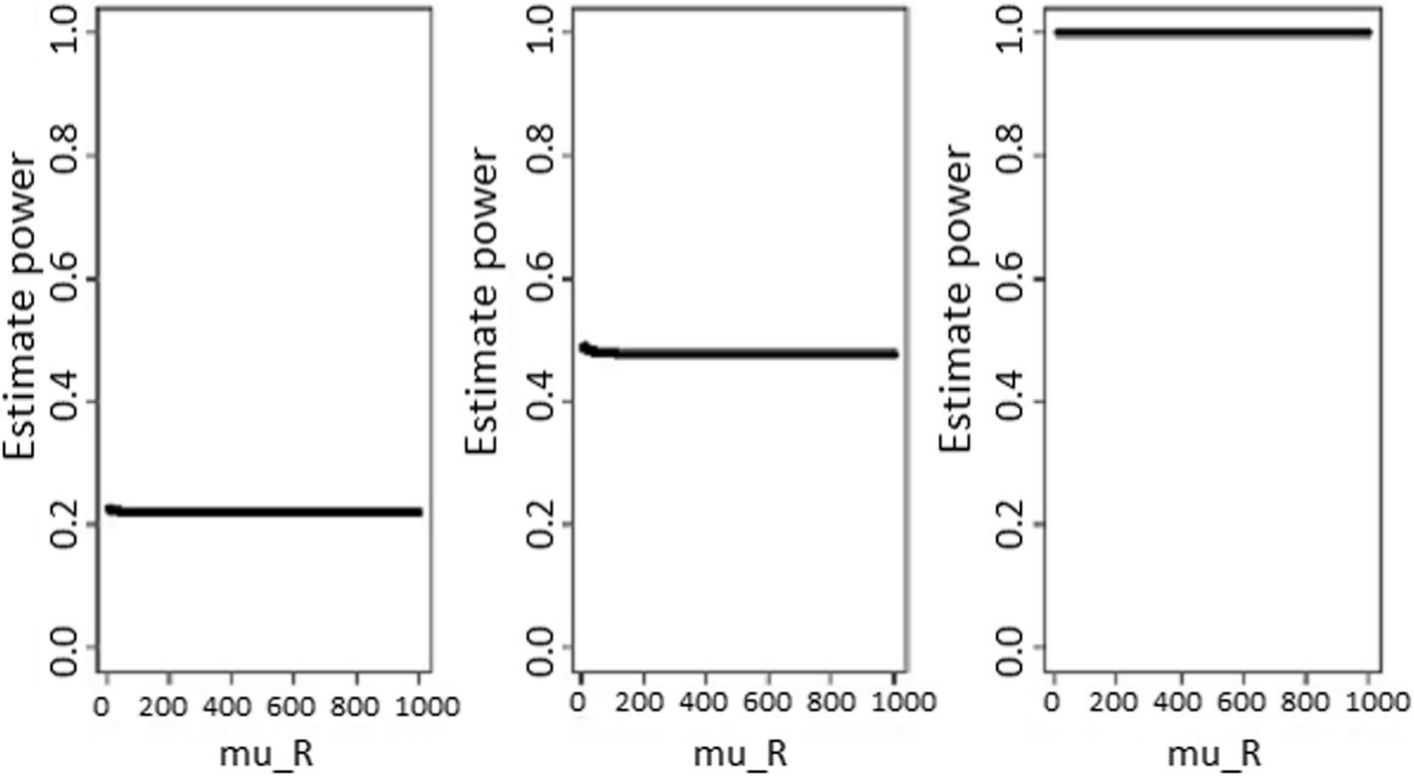
Power estimates according to sample sizes for test statistic based test (with standardized non-inferiority difference delta = 0.05). Power estimates as function of reference treatment (with standardized non-inferiority difference delta = 0.05), for test statistic based test. From the left to the rigth, sample sizes are *n*_*N*_=*n*_*R*_=20, 100, and 1000, respectively

**Fig. 3:**
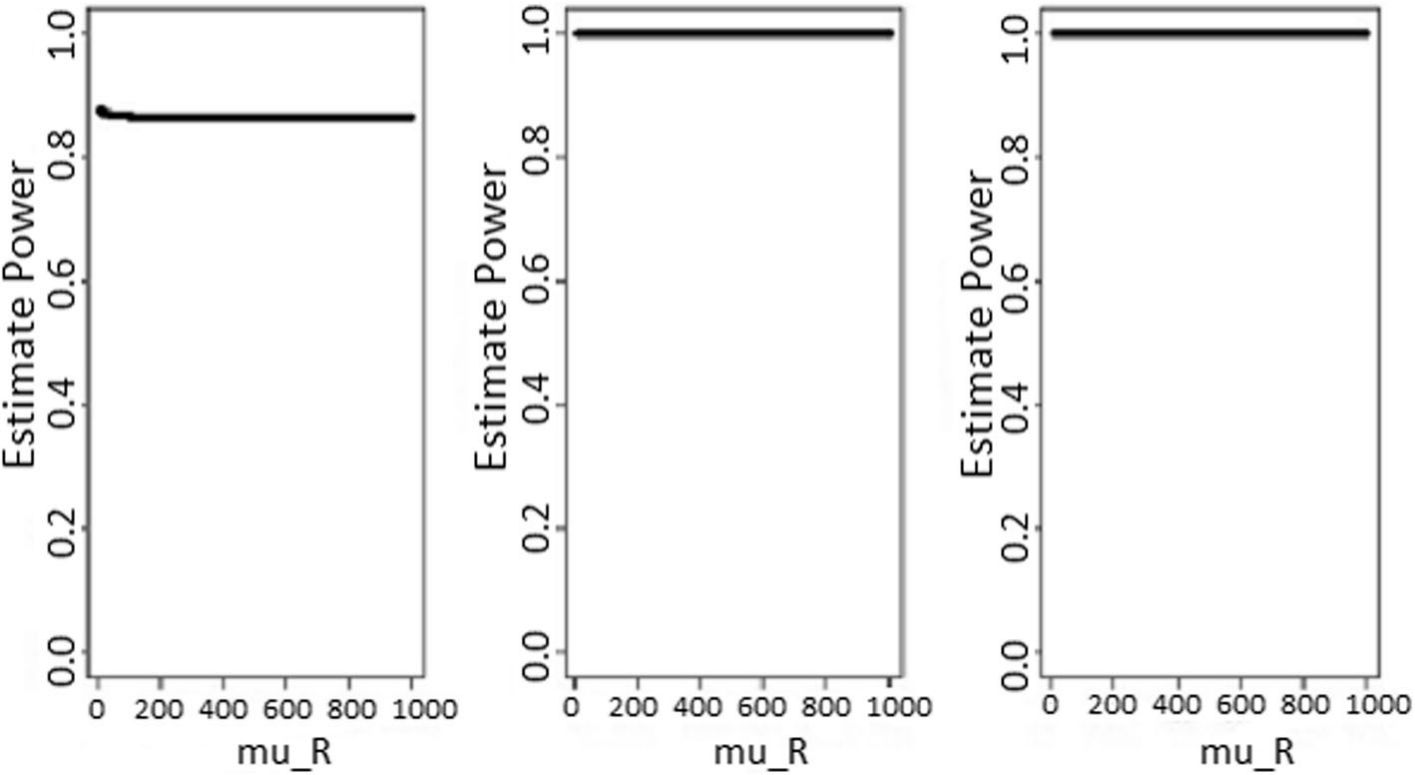
Power estimates according to sample sizes for test statistic based test (with standardized non-inferiority difference delta = 0.5). Power estimates as function of reference treatment (with standardized non-inferiority difference delta = 0.5), for test statistic based test. From the left to the rigth, sample sizes are *n*_*N*_=*n*_*R*_=20, 100, and 1000, respectively

The results for the approach based on a statistic are summarized in Figs. 1, 2, and 3 for type I error rate and power estimates, respectively. Whatever the sample size, it is observed that the type I error rate estimates were constant and were not *μ*_*R*_ dependent. For small sample size, the type I error rate estimate was slightly above the nominal value, while the median value estimate was 0.053, and an Interquartile Range(IQR) of [0.051−0.054]. As the sample size increases, the type I error estimates get close to the nominal value. In effect, for medium sample size of *n*=100, the type I error estimate is close to the nominal value, the median value estimate for *μ*_*R*_ was 0.051 (*IQR*=[0.050−0.052]). For large sample sizes, for example, *n*=1000, the type I error estimate was more accurate and closer to the nominal value, the median estimate was 0.050 (*IQR*=[0.050−0.050]).

The power estimates were summarized in Figs. 2 and 3, and they were not *μ*_*R*_-dependent. As expected, the power increased with sample sizes for fixed standardized non-inferiority difference *δ*, and larger values of *δ* led to a higher power estimate for fixed sample size.

#### Confidence interval based test

**Fig. 4:**
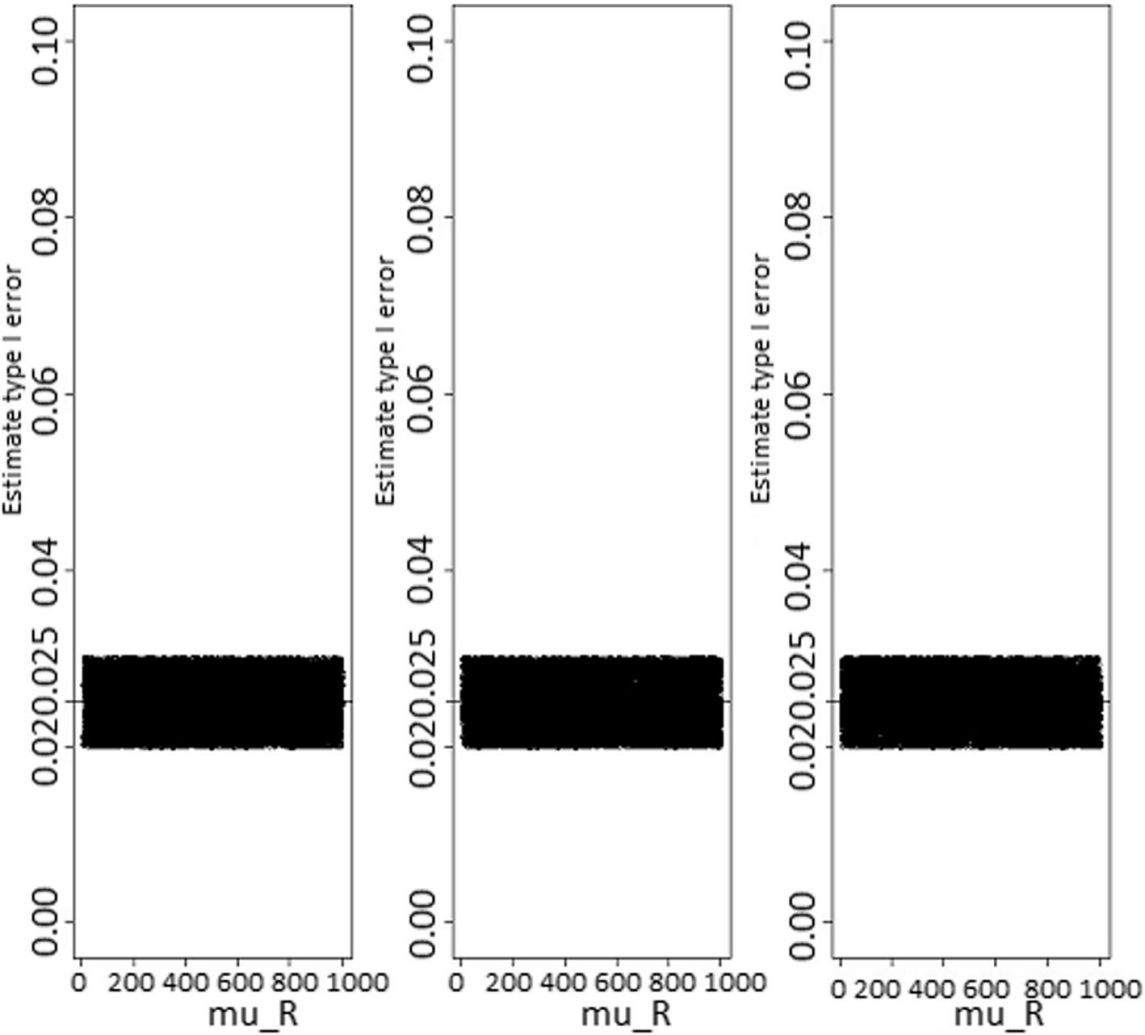
Type I error rate estimates according to sample sizes for the 95% one-sided confidence intervals level based test. Type I error rate estimate as function of reference treatment, for the 95% one-sided confidence intervals level based test. From the left to the rigth, sample sizes are *n*_*N*_=*n*_*R*_=20, 100, and 1000, respectively

**Fig. 5:**
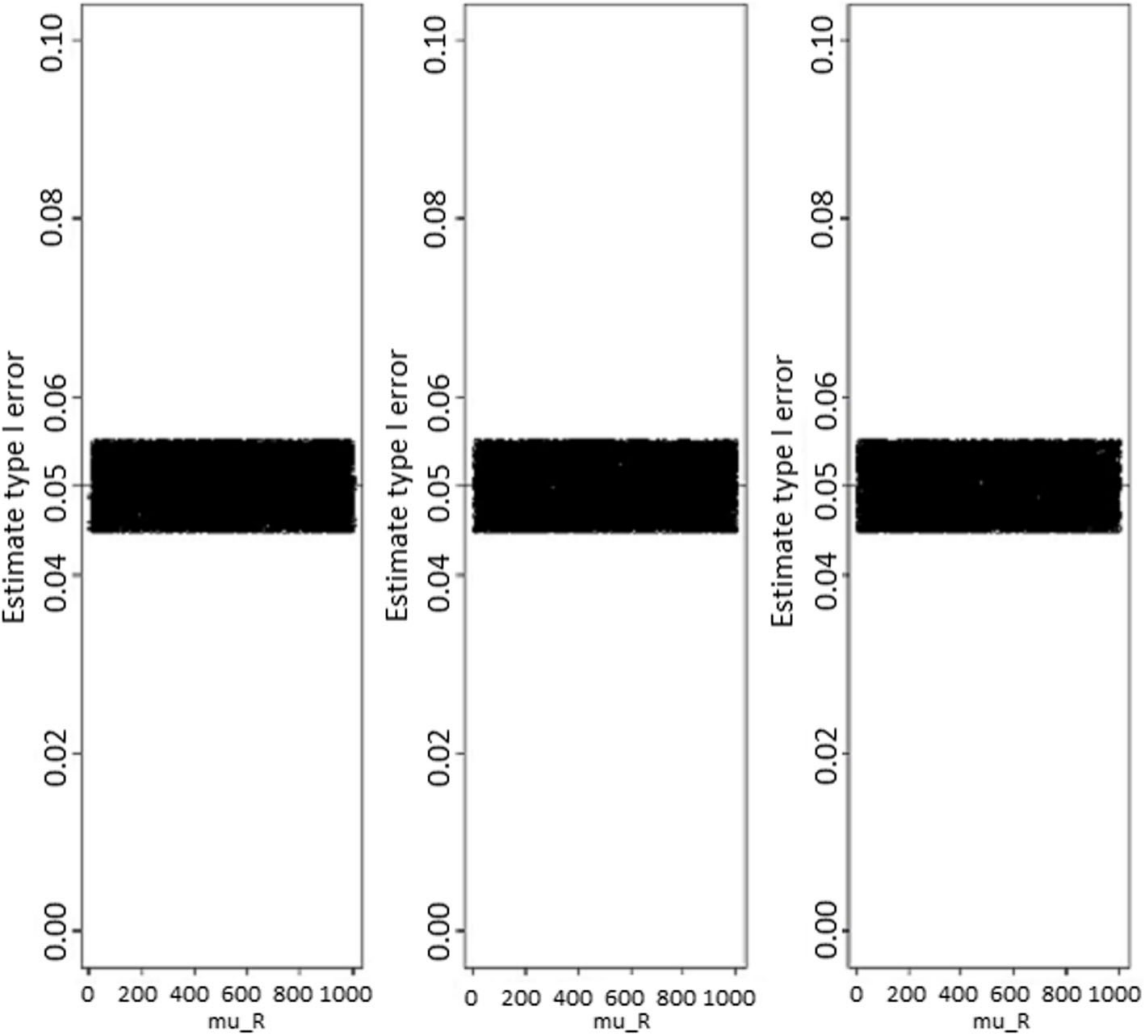
Power estimates according to sample sizes for the 95% one-sided confidence intervals level based test. Power estimates as function of reference treatment, for the 95% one-sided confidence intervals level based test. From the left to the rigth, sample sizes are *n*_*N*_=*n*_*R*_=20, 100, and 1000, respectively

**Fig. 6:**
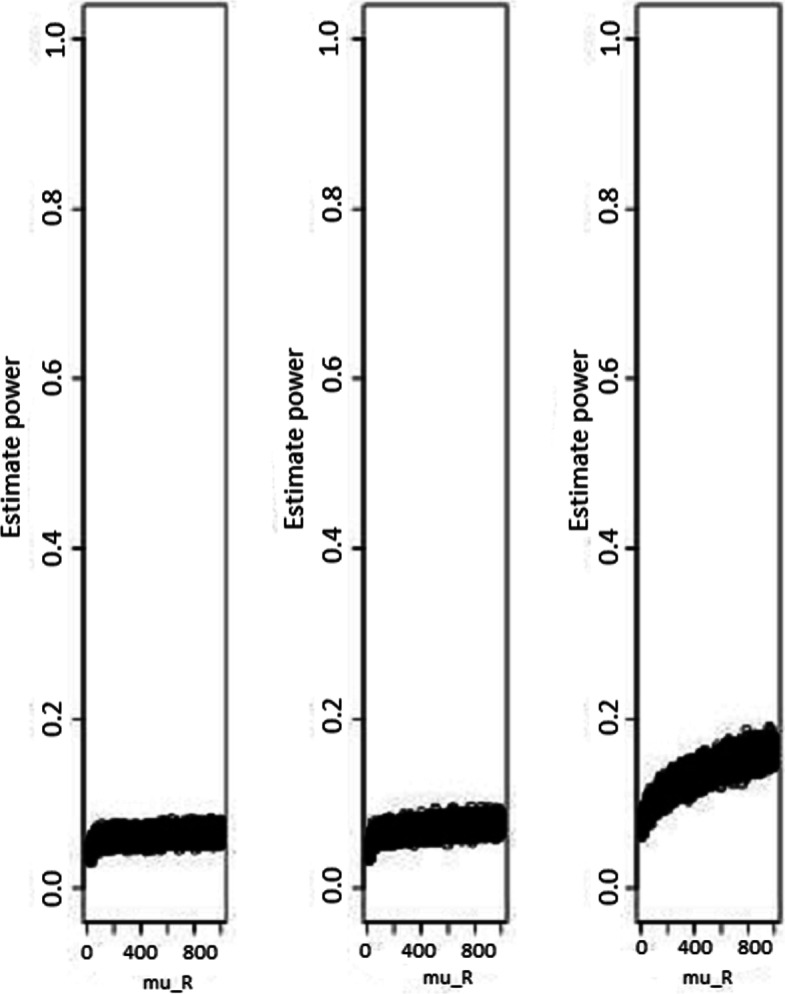
Type I error rate estimates according to sample sizes for the 95% two-sided confidence intervals level based test. Type I error rate estimate as function of reference treatment, for the 95% two-sided confidence intervals level based test. From the left to the rigth, sample sizes are *n*_*N*_=*n*_*R*_=20, 100, and 1000, respectively

**Fig. 7:**
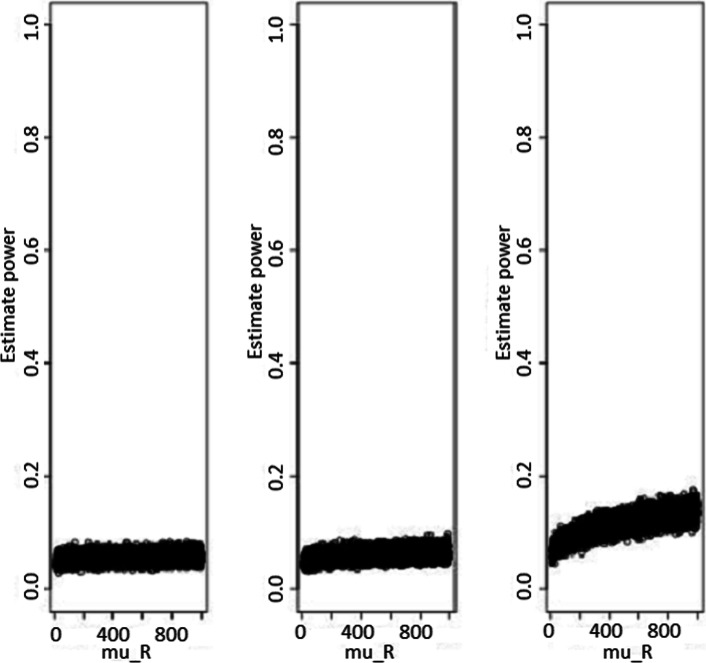
Power estimates according to sample sizes for the 95% two-sided confidence intervals level based test. Power estimates as function of reference treatment, for the 95% two-sided confidence intervals level based test. From the left to the rigth, sample sizes are *n*_*N*_=*n*_*R*_=20, 100, and 1000, respectively

The results for the approach based on confidence intervals are summarized in Figs. 4, 5, 6, and 7. For 95% both one- and two-sided confidence interval levels, the estimate type I error rates remained around 0.05 and 0.025, respectively, and are more concentrated around those values as the sample sizes get larger. Then, for a given nominal type I error of *α*, the suitable confidence intervals level would be 1−*α* and 1−2*α* for one- and two-sided confidence intervals, respectively. The power (at the equivalence point, *μ*_*R*_=*μ*_*N*_) increases with the sample sizes, but the convergence to 1 seemed to require very large sample sizes. This is not the case for the test statistic based method. Therefore, in terms of power estimate, the approach based on the test statistic would perform better than the confidence intervals based approach.

### The Stratall ANRS 12110 / ESTHER trial

The proposed methods were also applied to the Stratall ANRS 12110 / ESTHER tria, based on Observer and LOCF data, with a linear margin of $\Delta _{L}(R)= \frac {25}{100} R$. The results for the approach based on the test statistic are summarized in Table 1. The *p*-value is calculated based on the test statistic in Eq. (6). The statistical power was computed using Eq. (8) and based on the same inputs as in [17], which were *μ*_*N*_=*μ*_*R*_=140 and *σ*_*N*_=*σ*_*R*_=130. For the Observed data, the *p*-value estimate was =0.02, and the null hypothesis that CLIN was inferior to the LAB was rejected at 0.05 level. On the other hand, for the LOCF data, the *p*-value was =0.09, and the null hypothesis that CLIN was inferior to the LAB was not rejected at 0.05 level.

**Table 1:** *p*-value and power determination for the approach based on the asymptotic test statistic and according to the data used

	*p*-value	*Power*
Case of LOCF	0.02	0.77
Case of observed data	0.11	0.82

For the confidence interval-based approach, the test was performed by considering the one- and two-sided confidence interval levels. The results are presented in Table 2. The null hypothesis that CLIN was inferior to LAB was not rejected for any of the confidence intervals used with “LOCF data.” On the other hand, when using “Observed data,” the null hypothesis of inferiority was not demonstrated.

**Table 2:** Confidence interval calculations and decision on non-inferiority confidence interval based test

	One-sided CI	Two-sided CI
	Case of LOCF	
*CLIN*−*LAB*+*Δ*_*L*_(*LAB*)	− 5 to*∞*	− 10 to52
Decision	Inferiority	Inferiority
	Case of observed data	
*CLIN*−*LAB*+*Δ*_*L*_(*LAB*)	7 to*∞*	1 to72
Decision	Non-inferiority	Non-inferiority

The two proposed methods produced consistent results on the Stratall ANRS 12110 / ESTHER trial. Moreover, based on LOCF data, the obtained results are in line with those in [17]: the clinical monitoring alone was inferior to laboratory plus clinical monitoring.

## Discussions

In this study, we have proposed two non-inferiority test approaches for a continuous endpoints with flexible margins: a test based on a test statistic and a confidence interval based test. The confidence interval approach is more used in literature and recommended by the international guideline [2]. For the non-inferiority test with continuous endpoints and fixed margin, some studies like [7] and [12] studied the confidence interval approach which does not allowed for explicit sample size calculation. Comparatively, our proposed test based on a statistic allows explicit calculation of sample size and power formula.

The simulation results for the confidence intervals based test showed that the confidence interval level determined approximatively the type I error rate. The test with 95% one- and two-sided confidence intervals level led to type I errors which were approximated by 0.05 and 0.025, respectively. Therefore, for a given nominal type I error *α*=0.05, the confidence intervals based test would be performed with one- or two-sided confidence intervals with 1−*α* or 1−2*α* levels, respectively; these findings are consistent with those in [7]. The non-inferiority hypothesis test is a one-tailed test, so when performing the testing procedure with the classical nominal type I error *α*, the actual type I error would be *α*/2. Therefore, for a given desired nominal type I error, to avoid the conservativeness of the test, the test should be performed with this nominal error times two. However, the debate on which of the one- or two-sided confidence intervals should be used in non-inferiority trials remains open, which is discussed in [20].

The most important output of this study was the type I error which was not varying according to the value of reference treatment, either for the test based on a statistic or the test based on confidence intervals. This suggested that the variability and uncertainty around the margin were accounted for, without affecting the properties of the proposed tests. The proposed methods in this study could therefore be viewed as a generalization of the case where the non-inferiority margin is fixed for continuous endpoints.

## Conclusions

In an active controlled trial of non-inferiority, the non-inferiority margin should be a function of reference treatment to account for the uncertainty surrounding the mean estimate of reference treatment. This paper produced a framework on how to perform the non-inferiority hypothesis test with a flexible margin. Based on type I one error rate and power estimates, the proposed non-inferiority hypothesis test procedures have good performances and are applicable in practice, a practical application on clinical data was illustrative.

## Data Availability

The datasets used and analyzed during the current study are available from the corresponding author or the author named Christian Laurent (christian.laurent@ird.fr) on reasonable request.

## Abbreviations

CD4: Cluster of differentiation 4
CLIN: Clinical monitoring alone
HIV/AIDS: Human immunodeficiency virus infection and acquired immune deficiency syndrome
LAB: Laboratory and clinical monitoring
LOCF: Last observation carried forward
